# Mining of key genes for cold adaptation from *Pseudomonas fragi* D12 and analysis of its cold-adaptation mechanism

**DOI:** 10.3389/fmicb.2023.1215837

**Published:** 2023-07-06

**Authors:** Changjie Bao, Muzi Li, Xuhui Zhao, Jia Shi, Yehui Liu, Na Zhang, Yuqi Zhou, Jie Ma, Guang Chen, Sitong Zhang, Huan Chen

**Affiliations:** ^1^Key Laboratory of Straw Comprehensive Utilization and Black Soil Conservation, Ministry of Education, Changchun, China; ^2^College of Life Science, Jilin Agricultural University, Changchun, China; ^3^Key Laboratory of Mollisols Agroecology, Northeast Institute of Geography and Agroecology, Chinese Academy of Sciences, Changchun, China

**Keywords:** psychrotrophs, cold-adaptation mechanism, genomics, transcriptomics, *Pseudomonas fragi*

## Abstract

The psychrotroph *Pseudomonas fragi* D12, which grew strongly under low temperatures, was screened from tundra soil collected from the permanent alpine zone on Changbai Mountain. To mine the genes critical for cold tolerance and to investigate the cold-adaptation mechanism, whole-genome sequencing, comparative genomic analysis, and transcriptome analysis were performed with *P. fragi*. A total of 124 potential cold adaptation genes were identified, including nineteen unique cold-adaptive genes were detected in the genome of *P. fragi* D12. Three unique genes associated with pili protein were significantly upregulated at different degrees of low temperature, which may be the key to the strong low-temperature adaptability of *P. fragi* D12. Meanwhile, we were pleasantly surprised to find that *Pseudomonas fragi* D12 exhibited different cold-adaptation mechanisms under different temperature changes. When the temperature declined from 30°C to 15°C, the response included maintenance of the fluidity of cell membranes, increased production of extracellular polymers, elevation in the content of compatibility solutes, and reduction in the content of reactive oxygen species, thereby providing a stable metabolic environment. When the temperature decreased from 15°C to 4°C, the response mainly included increases in the expression of molecular chaperones and transcription factors, enabling the bacteria to restore normal transcription and translation. The response mechanism of *P. fragi* D12 to low-temperature exposure is discussed. The results provide new ideas for the cold-adaptation mechanism of cold-tolerant microorganisms.

## 1. Introduction

Earth has an extremely complex eco-environmental system, comprising a variety of ecological communities in different climatic environments. However, more than three-quarters of the planet’s surface is in year-round low-temperature environments (e.g., alpine, deep sea, glacial, polar, cavernous, and artificial cold environments), often accompanied by other extreme conditions, such as high salinity, acidic, or anaerobic conditions (Margesin and Collins, 2019). Therefore, microorganisms that grow in a year-round low-temperature environment usually exhibit additional extreme tolerances (e.g., salt–alkali resistance, high radiation tolerance, or extreme anaerobicity), and thus have strong potential for biotechnological applications (Chevalier et al., 2000; Li et al., 2014; Zheng et al., 2020; Sun et al., 2021). Cold-tolerant microorganisms are mainly classified into two categories: psychrophiles (obligate psychrophiles) and psychrotrophs (facultative psychrophiles). Psychrophilic microorganisms are those whose maximum growth temperature does not exceed 20°C, can grow well at 0°C, and whose optimum growth temperature does not exceed 15°C; psychrotrophs are predominantly microorganisms that can grow and reproduce under the temperature range of 0–15°C with a maximum temperature tolerance of 20–25°C (Morita, 1975). Among these two groups, psychrotrophs have received more attention in practical production applications because of their advantages in adapting to temperature changes and the utilization of nutrients in the environment. To date, cold-tolerant microorganisms have been widely used in sewage treatment, straw returning, biological composting, and food and drug processing, in addition to other fields (Johnson et al., 1992; Whyte et al., 1998; Churchill et al., 1999; Nichols et al., 1999; Russell and Nichols, 1999; Margesin and Schinner, 2001). Therefore, studying the growth and metabolism of microorganisms in a low-temperature environment has important practical relevance.

Investigation of the cold-adaptation mechanism of cold-tolerant microorganisms is mainly focused on the following aspects. (i) Cell membrane fluidity: the cell membrane provides a stable environment for various physiological activities and the metabolism of microorganisms, and a change in cell membrane fluidity is the primary signal for cold-tolerant microorganisms to perceive cold stimuli (Tribelli and Lopez, 2018). When the temperature declines, the fluidity of the cell membrane decreases, thus affecting the normal physiological functioning of the membrane and the microorganism. Cold-tolerant microorganisms mainly adjust the fluidity of cell membranes by changing the composition of membrane lipids, such as increasing the proportion of unsaturated fatty acids, shortening the average chain length of fatty acid, increasing the proportion of *cis* fatty acids and hydroxy fatty acids, and reducing the proportion of cyclic fatty acids, to prevent the cell membrane from changing from a liquid crystal state to a gel state, thereby maintaining the fluidity of the cell membrane (Chintalapati et al., 2004; Chattopadhyay, 2006; Kloska et al., 2020). (ii) Cold shock proteins and helicases: when the ambient temperature decreases, microorganisms rapidly and abundantly induce the production of a variety of cold shock proteins, usually composed of 65–75 amino acids, with a conserved domain containing five antiparallel β-sheets, which form a β-barrel lamellar structure (Czapski and Trun, 2014). Cold shock proteins act together with RNases on entangled RNAs under cold stimulation, unwinding entangled RNAs and allowing microorganisms to resume normal transcription and translation, which is consistent with one aspect of the function of helicases (Hunger et al., 2006; Phadtare, 2012; Zhang et al., 2018). (iii) Psychrophilic enzymes: these enzymes can only maintain high catalytic efficiency at relatively low temperatures, and are quickly inactivated at slightly higher temperatures, thus showing high thermal sensitivity. In a low-temperature environment, enzymatic reactions are performed through psychrophilic enzymes to maintain normal metabolism (Mandelman et al., 2001; Parveen et al., 2019). (iv) Extracellular polymers: these are a type of macromolecular polymer secreted by microorganisms that adhere to the surface of microorganisms and the surrounding environment during the formation of biofilms. The polymer is mainly composed of polysaccharides and proteins, in addition to a small number of nucleic acids. As a cryoprotectant, extracellular polymers can reduce the freezing point of the environment around the microorganism, promote surface adhesion, cell aggregation, and biofilm formation, and can also protect extracellular enzymes from low-temperature deformation, thereby ensuring that microorganisms have a stable metabolic environment under low temperatures (Kelley et al., 1997; Williams et al., 2009; Caruso et al., 2018). (v) Compatible solutes: solutes such as glycine, betaine, choline, glycerol, trehalose, mannitol, and sorbitol can accumulate to a high concentration in the cytoplasm without disrupting cellular activities and can change the microstructure of ice crystals, protecting the cell from damage from low temperature (Hoffmann and Bremer, 2011; Wu et al., 2013; Manzanera, 2021). (vi) Reactive oxygen species (ROS) balance: given the increased solubility of oxygen at low temperatures, the ROS concentration increases, which can cause severe oxidative damage to cells (Médigue et al., 2005). Therefore, cold-tolerant microorganisms produce antioxidant enzymes, such as catalase and superoxide dismutase, to prevent oxidative damage (Giordano et al., 2020).

*Pseudomonas* is a typical cold-tolerant microorganism and can produce a variety of extracellular enzymes, including lipase, protease, and amylase. To date, a variety of *Pseudomonas* isolates have been used in industrial production, environmental management, and agricultural production (Kelley et al., 1997; Mandelman et al., 2001; Médigue et al., 2005; Williams et al., 2009; Hoffmann and Bremer, 2011; Wu et al., 2013; Li et al., 2014; Caruso et al., 2018; Parveen et al., 2019; Sandini et al., 2019; Giordano et al., 2020; Phukon et al., 2020; Zheng et al., 2020; Manzanera, 2021; Sun et al., 2021). *Pseudomonas fragi* has received extensive attention owing to its responsibility for spoilage of certain food products. For example, Wu Q. et al. (2022) found that *P. fragi* EH-H1 can effectively carry out heterotrophic nitrification and aerobic denitrification at 15°C, proving that it has outstanding low-temperature denitrification ability, which can help low-temperature wastewater treatment. Wu screened and obtained an excellent strain P. fragi NL20W that can efficiently utilize waste whey to produce lactobionic acid. At the same time, he found a new lactose oxidase by whole-genome sequencing. After heterologous expression by *P. putida* KT2240, lactose content increased by 486.1%, which laid the foundation for the further use of metabolic engineering to increase the production of lactobionic acid (Wu J. et al., 2022). Avcı used *P. fragi* as a biocatalyst, combined with graphene-Au/CFE, developed and optimized the electrode material, and prepared a microbial fuel cell (Avcı et al., 2021). Awolope discovered the potential of *P. fragi* A13BB as a rhizosphere microorganism through whole genome sequencing analysis, proving its value in agricultural production (Awolope et al., 2021). Numerous studies have demonstrated the potential application value of *P. fragi* in many fields, but few studies have been conducted on its low-temperature adaptation mechanism and specific application at low temperatures. In a preliminary study, we used soil collected from the perennial alpine region of Changbai Mountain as the bacterial source material and screened a large number of cold-tolerant microorganisms. Comparison of the growth characteristics of the isolates revealed that one strain grew significantly faster than all other strains at 15°C. The isolate had simple nutritional requirements, could utilize a variety of carbon and nitrogen sources for growth, and produced protease, amylase, and other extracellular enzymes. The isolate had favorable application potential and subsequently was identified as *Pseudomonas fragi* and designated strain D12. In the present study, the crucial genes associated with cold adaptation of *P. fragi* D12 were investigated using comparative genomics and transcriptomics. Our aim is to elucidate the cold-adaptation mechanism of the strain under perennial low-temperature stress and to provide a reference for the regulation of the microbial response to low-temperature stress.

## 2. Experimental procedures

### 2.1. Strain screening

Soil samples (5 g) collected from the perennial alpine region of Changbai Mountain (41°99′41”N, 128°02′36″E; Jilin, China) were placed in a sterile triangular flask containing glass beads. Sterile water (100 mL) was added and the flask was initially shaken vigorously, and then shaken at 180 rpm for 10 min at 10°C. Next, 5 mL of the enriched soil solution was diluted with 45 mL sterile water, mix well and allowed to stand for 30 min. Four dilution gradients (10^−1^, 10^−2^, 10^−3^, and 10^−4^) were prepared with sterile water. An aliquot (100 μL) of each dilution was spread evenly on tryptic soy broth (TSB) solid medium and incubated in a 10°C incubator. Based on the colony size, color, glossiness, edge shape, and other indicators, each isolate was subjected to further streak culture and purified to obtain cold-tolerant bacterial isolates. One percent of the inoculum was added to 100 mL TSB liquid medium and the OD_600_ was measured every 2 h to generate a growth curve. The strain with the fastest growth rate and the largest growth amount was selected as a model cold-tolerant strain for subsequent experiments. After screening, the strains were stored in −80°C ultra-low temperature refrigerator.

### 2.2. Strain identification

The growth characteristics of the strains were evaluated by measuring the OD_600_ under culture at different temperatures (4, 10, 15, 20, 25, and 37°C) every 2 h. The species traits of strain D12 were assessed by investigating the morphology, physiological, and biochemical characteristics, and molecular biological information. The color, shape, size, and surface morphology of the strains were observed under a microscope (ZEISS Axio Vert A1 and Philips XL30 ESEM). Physiological and biochemical tests included the Voges–Proskauer reaction, methyl red test, starch hydrolysis test, 7% sodium chloride growth test, catalase test, and sugar and alcohol fermentation test (Rehman et al., 2015). Total genomic DNA of strain D12 was extracted using the TIANamp Bacteria DNA Kit (DP302-02, TIANGEN, China). The 16S rDNA gene was amplified by PCR and sequenced by the Shanghai Sangon Bioengineering Co., Ltd. The specific primer information is as follows: F: AGTTTGATCMTGGCTCAG; R: GGTTACCTTGTTACGACTT. The sequences were submitted to the GenBank database (OP535634) and used as the query for a BLAST search of the GenBank database for similar sequences. The 16S rDNA of the strain with the highest similarity was selected, and a dendrogram was constructed using the neighbor-joining method with MEGA software (nucleotide sequences).

### 2.3. Strain activation and genome extraction

The strain D12 stored in a − 80°C ultra-low-temperature freezer (EXF24086V, Thermo Fisher Scientific, USA) was thawed on ice, streaked on Lysogeny broth (LB) solid medium, and cultured in a 25°C incubator for 14–16 h. After subculture 2–3 times, a single colony was inoculated into 100 mL LB liquid medium and shaken at 180 rpm for 14 h at 30°C (OD_600_ = 1.0–1.2).

Total genomic DNA of strain D12 was extracted using the TIANamp Bacteria DNA Kit (TIANGEN) in accordance with the manufacturer’s instructions. The quality of extracted DNA was checked with an ultra-micro nucleic acid protein analyzer (ScanDrop100, Analytik Jena, Germany) and agarose gel electrophoresis.

### 2.4. Whole-genome sequencing and splicing of strains

The genomic DNA of strain D12 was submitted for whole-genome sequencing using PacBio RS II and Illumina HiSeq 4,000 platforms by the Beijing Genome Research Institute (Shenzhen, China). The PacBio Sequel platform uses the Zero-Mode Waveguides sequencing array of 4 SMRT cells to generate subreads, which were subject to data processing to filter out low-quality sequences and adapter sequences. The subreads were corrected with Pbdagcon software^1^ to obtain corrected reads with high reliability, and the Celera Assembler^2^ was used to assemble the high-quality corrected reads to obtain the optimal assembly. Finally, to improve the accuracy of the genome sequence, the assembly was single-base corrected using the GATK^3^ and SOAP toolkits (SOAP2, SOAPsnp, and SOAPindel) to distinguish the genome sequence from the plasmid sequences (Klemm and Christiansen, 1990; Eid et al., 2009; Kim et al., 2014; Utturkar et al., 2014).

### 2.5. Analysis of strain genome components

Predictive analysis of the genome composition of strain D12 was performed using a hidden Markov model with Glimmer software^4^ (Delcher et al., 1999, 2007). Prediction of rRNA, tRNA, and sRNA sequences was conducted with RNAmmer software,^5^ tRNAscan software,^6^ and Rfam software,^7^ respectively. Tandem repeat sequences were predicted with TRF software^8^ (Lowe and Eddy, 1997; Benson, 1999; Lagesen et al., 2007; Gardner et al., 2009). Microsatellite and minisatellite sequences were screened out according to the length and number of repeat units. Gene islands were predicted using methods such as IslandPath-DIOMB, SIGI-HMM, and IslandPicker with the Genomic Island Suite of Tools.^9^ Pre-phage regions were predicted using the PHAge Search Tool^10^ and CRISPRs were identified using CRISPRCasFinder software^11^ (Couvin et al., 2018).

### 2.6. Gene annotation and protein classification

Gene annotation was mainly based on alignment of amino acid sequences. The BLAST alignment tool was used to align the amino acid sequence of the gene with selected databases to obtain the corresponding functional annotation information and retain the optimal alignment as the gene annotation. Seven databases were used for general gene function annotation, namely the KEGG (Kyoto Encyclopedia of Genes and Genomes), GO (Gene Ontology), COG (Cluster of Orthologous Groups of proteins), NR (Non-Redundant Protein Database), Swiss-Prot, TrEMBL, and EggNOG databases. Four databases were used for analysis of bacterial pathogenicity and drug resistance, namely the VFDB (Virulence Factors of Pathogenic Bacteria), ARDB (Antibiotic Resistance Genes Database), PHI (Pathogen Host Interactions), and CAZy (Carbohydrate-Active enZYmes) databases (Ashburner et al., 2000; Liu and Pop, 2009; Levasseur et al., 2013; Galperin et al., 2015; Chen et al., 2016; Huerta-Cepas et al., 2016; Kanehisa et al., 2016). The type III secretion system effector protein was predicted using Effective T3.

### 2.7. Comparative genomics and phylogenetic analysis

To analyze the gene structure and gene characteristics of strain D12, five strains of *P. fragi* (NMC25, NL20W, DBC, NRRLB727, and A13BB) with the closest homology to strain D12 were selected in NCBI genome database. Whole genomes were used as the reference sequence genomes for comparative genomic analysis. The genome sequence of strain D12 was sorted according to the genome sequence of the reference bacteria based on the alignment results generated with MUMmer^12^ and core- and pan-genome calculations with BLAST. Similar proteins were clustered with CD-HIT rapid clustering software^13^ (Fu et al., 2012). Gene families were constructed for all genes of the tested strains and were analyzed using multiple software: after BLAST alignment of the protein sequences, Solar was used to remove redundancy, and Hcluster_sg software was used to compare the results to perform TreeFam clustering of gene families. A dendrogram for the gene families was constructed using the neighbor-joining method with Treebest software^14^ (Muscle, 2004; Nandi et al., 2010).

### 2.8. Transcriptome sample processing and sequencing

A single colony of strain D12 was inoculated into 100 mL LB liquid medium, cultured at 160 rpm at 30°C to OD_600_ = 1.0, and cultured at 30°C, 15°C, or 4°C for 2 h, and recorded as Group 30 (G30), Group 15 (G15), and Group 4 (G4), respectively. Three replicates per treatment group were performed. The TRNzol Universal Reagent (DP424, TIANGEN) was used to extract bacterial RNA, which was sequenced by the Suzhou Genewiz Biotechnology Co., Ltd. (Suzhou, China). To remove technical sequences, including adapters, PCR primers, or fragments thereof, and low-quality sequences of less than 20 bases, the data set in fastq format was processed by Cutadapt (Martin, 2011). First, the reference genome sequences and gene model annotation files of related species were downloaded from genome databases, such as UCSC, NCBI, and ENSEMBL. Second, Bowtie2 (v2.2.6) was used to index the reference genome sequence. Finally, clean data were aligned to the reference genome with Bowtie2 (v2.2.6) (Langdon, 2015).

### 2.9. Transcriptome data analysis

The transcripts data in fasta format was converted from the known gff annotation file and indexed properly. Using the file as a reference gene file, HTSeq (v0.6.1p1) was used to estimate gene expression levels from the paired-end clean data (Anders et al., 2015). Differential expression analysis was conducted with the DESeq2 Bioconductor package for R using a model based on the negative binomial distribution (Anders and Huber, 2010). After adjustment using Benjamini and Hochberg’s approach for controlling the false discovery rate, differentially expressed genes (DEGs) were detected based on the criterion *P*_adj_ < 0.05 and the log2 of the expression changes by more than twice. GOSeq (v1.34.1) was used to identify gene ontology (GO) terms significantly enriched among the genes (*value of p* <0.05). The topGO R package was used to visualize the enrichment results (Kanehisa and Goto, 2000; Tatusov et al., 2000; Young et al., 2010).

Rockhopper uses a Bayesian approach to create a transcriptome map including transcription start/stop sites for protein-coding genes and novel transcripts identified by Rockhopper. Samtools v0.1.19 with the command mpileup and Bcftools v0.1.19+ were used to perform single-nucleotide variant calling (Ramirez-Gonzalez et al., 2012; Etherington et al., 2015; Danecek et al., 2021). Rockhopper (2.0.3) was employed to predict operons, transcription start sites, and transcription stop sites (Waldoch et al., 2012). RBSfinder (v1.0) was used for Shine–Dalgarno sequence prediction (Florensa et al., 2022). TransTermHP (v2.09) was used to accurately detect Rho-independent transcription terminators. Intergenic novel transcripts were searched against the NR database with BLAST; non-annotated transcripts were considered to be potential *trans*-encoded sRNAs. Novel antisense transcripts were treated as *cis*-encoded sRNAs. The secondary structures of the sRNA were predicted using RNAfold (v2.3.2) (Denman, 1993).

## 3. Results and discussion

### 3.1. Strain screening and identification

**Figure 1:**
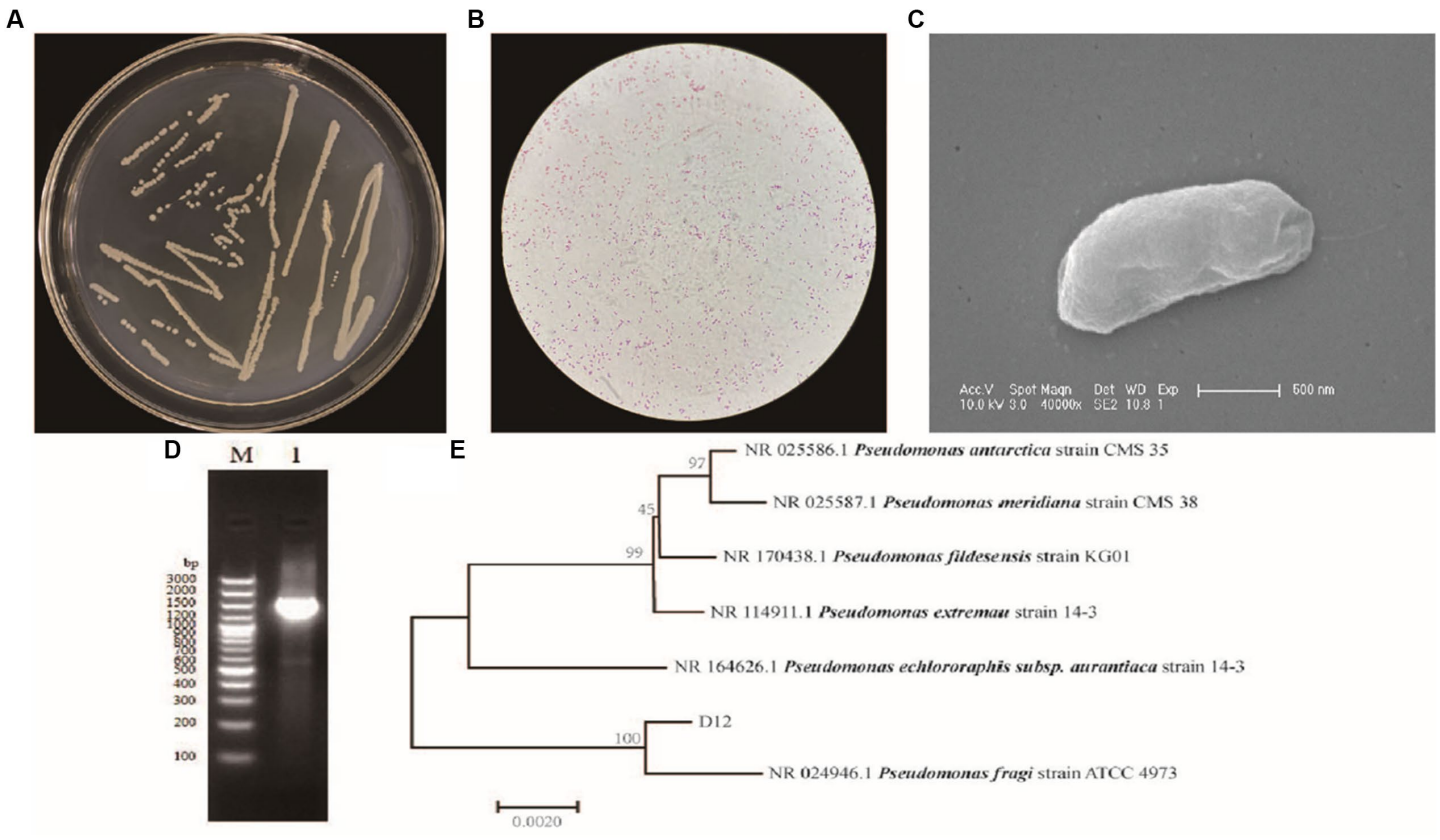
Morphological and molecular biological identification for *Pseudomonas fragi* D12. **(A)** Colony morphology; **(B)** gram staining (optical microscope 400×); **(C)** scanning electron micrograph (40,000×); **(D)** 16 s rDNA electropherogram and sequence; **(E)** neighbor-joining dendrogram for the 16S RNA gene of *Pseudomonas* strains.

Comparison of the growth characteristics of the cold-tolerant bacterial isolates revealed that the growth rate of strain D12 was significantly faster than that of other strains at low temperature and the increase in population size was the greatest. The colonies of strain D12 were round, white, opaque, raised in the center, and smooth at the edges (Figure 1A). Gram staining was negative (Figure 1B). The cells were short, rod-shaped, and approximately 0.5 μm × 2 μm (Figure 1C). The physiological and biochemical indicators for strain D12 are summarized in Table 1. The strain D12 grew in 7% sodium chloride medium, the Voges–Proskauer test was positive, and the methyl red test was negative, indicating that the strain decomposed glucose to produce pyruvate. After further decomposition, acidic products, such as lactic acid, succinic acid, acetic acid, and formic acid, were not produced. The catalase test was positive, indicating that the strain produced catalase. The sugar and alcohol fermentation experiments showed that strain D12 could ferment glucose, sucrose, maltose, starch, mannitol, and other sugars to produce acids, but the utilization capacity varied: the ability to utilize glucose was strongest, followed by maltose and starch, and the ability to utilize sucrose and mannitol was weakest. Based on *Bergey’s Bacterial Identification Manual*, the strain was preliminarily identified as an isolate of *Pseudomonas*. Construction of a dendrogram based on 16S rDNA sequences indicated that strain D12 was most similar to *P. fragi* ATCC4973 (Figure 1E). Based on the morphological observations in combination with the physiological and biochemical test results, strain D12 was identified as *P. fragi* and designated *P. fragi* D12.

**Table 1:** Physiological and biochemical indicators for *Pseudomonas fragi* D12.

Project	Result	Project	Result
7% Sodium chloride growth	−	V - P test	++
Methyl red test	−	Catalase test	+
Starch hydrolysis test	+	Sugar and alcohol fermentation experiment - Glucose	+++
Sugar and alcohol fermentation experiment - Sucrose	+	Sugar and alcohol fermentation experiment - Maltose	++
Sugar and alcohol fermentation experiment - Starch	++	Sugar and alcohol fermentation experiment - Mannitol	+

“+++” indicates a strong positive result, “++” indicates a positive result, “+” indicates a weakly positive result, “−” indicates a negative result.

### 3.2. Genome analysis

**Figure 2:**
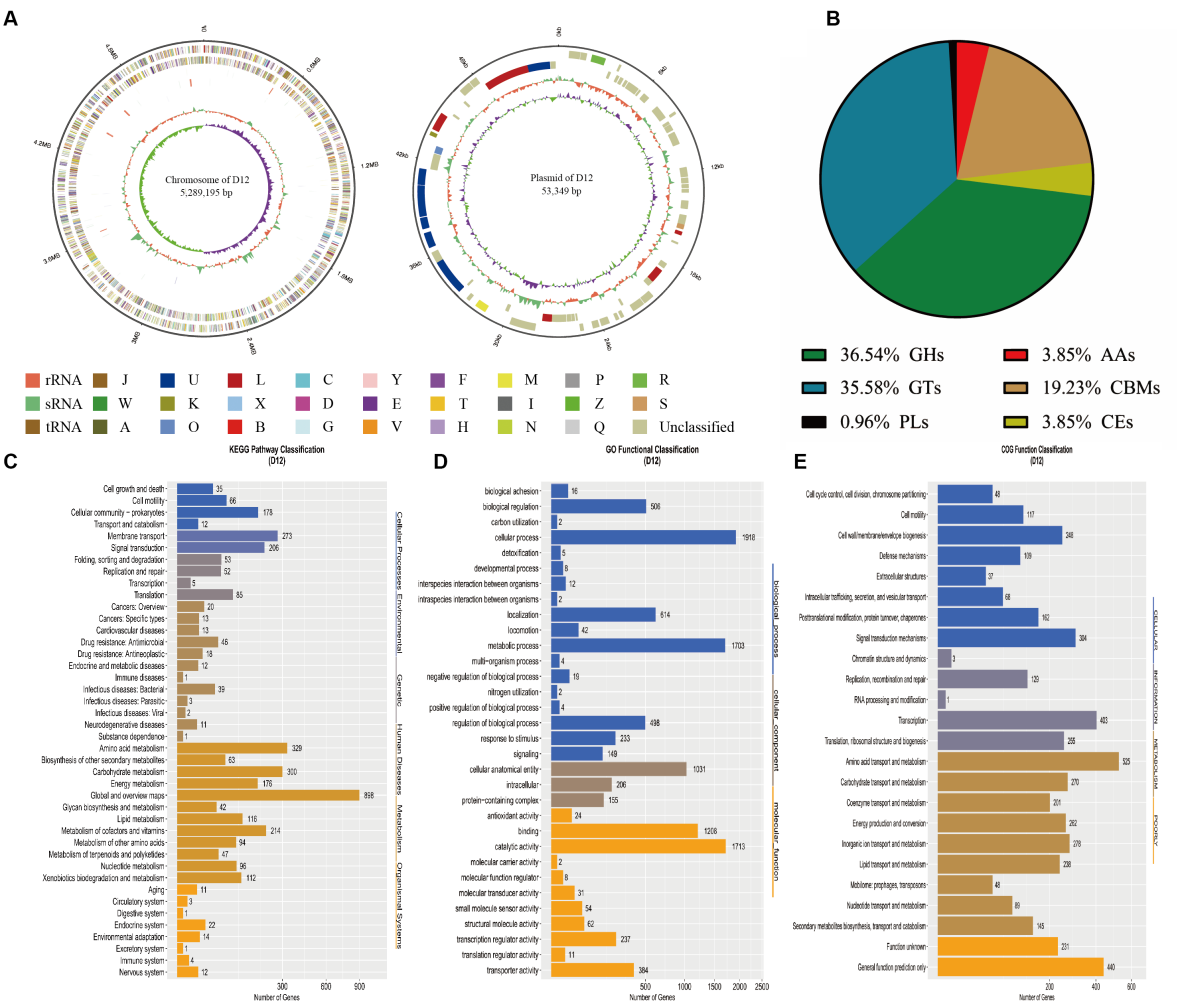
Genomic information and gene annotation for *Pseudomonas fragi* D12. **(A)** Chromosome circle diagram (left) and plasmid circle diagram (right). The seven circles in the chromosome circle diagram are as follows (from outer to inner): genome size; leading strand (the coding genes are classified and colored according to the COG database); lagging strand (the coding genes of are classified and colored according to the COG database); ncRNA of the leading strand; ncRNA of the lagging strand; GC content; and GC offset (green is negative, purple is positive). The five complete circles in the plasmid circle diagram are as follows (from outer to inner): genome size; leading strand (coding genes are classified and colored according to the COG database); lagging strand (coding genes are classified and colored according to the COG database); GC content; and GC offset. **(B)** CAZy functional classification; **(C)** KEGG pathway classification; **(D)** GO functional classification; **(E)** COG functional classification.

Whole-genome sequencing generated a total of 8,764,210 read-length fragments. After splicing and alignment analysis, the genome of *P. fragi* D12 was composed of a single-stranded circular chromatogram and a circular plasmid (Figure 2A). Basic genomic information is presented in Table 2. The full-length chromosome sequence was 5,289,195 bp and the GC content was 57.36%. The full-length plasmid sequence was 53,349 bp and the GC content was 54.95%. Glimmer software predicted 4,860 genes with an average length of 952.16 bp. Among the non-coding RNAs, 69 tRNAs, nine 5S rRNAs, eight 16S rRNAs, eight 23 s rRNAs, and 35 sRNAs were predicted. In addition, 76 tandem repeats, including 37 minisatellite DNAs and 19 microsatellite DNAs, were predicted. CRISPR was 140 bp in length in the plasmids and 205 bp in length in the chromosomes.

**Table 2:** Basic genomic information for *Pseudomonas fragi* D12.

Attributes	Number
Chromosome length (bp) and GC content (%)	5,289,195 / 57.36%
Plasmid length (bp) and GC content (%)	53,349 / 54.95%
tRNA	69
5 s rRNA	9
16 s rRNA	8
23 s rRNA	8
sRNA	35
Tandem repeat	76
Minisatellite DNA	37
Microsatellite DNA	19
Prophage	7
Virulence factors	440
Drug resistance gene	26
Type III secretion system effector protein	679

Prophages are nucleic acids of mild phages integrated into the host genome. The presence of prophage sequences may enable bacteria to acquire antibiotic resistance, enhance adaptability to the environment, and improve adhesion. Seven prophage sequences were detected in the genome of *P. fragi* D12 of which six were in the chromosomes and one in the plasmids. With regard to bacterial pathogenicity and drug resistance, a total of 440 virulence factors, 26 drug resistance genes, and 679 type III secretion system effector proteins were predicted. With consideration of the complete genome size, the proportion was small, which indicated that *P. fragi* D12 is not a harmful pathogen and may have potential for specific biological applications.

The CAZy database predicted a family of related enzymes that catalyze carbohydrate degradation, modification, and biosynthesis. The family mainly included glycoside hydrolases (GHs), glycosyl transferases (GTs), polysaccharide lyases (PLs), carbohydrate esterases (CEs), and auxiliary activities (AAs). In addition, carbohydrate-binding modules (CBMs) were detected. The GHs and GTs accounted for a large proportion of the genome (Figure 2B). This may provide a basis for the strain to survive in different environments, which is an important factor for the strain to survive in extremes, and also may be associated with the production of a variety of extracellular enzymes by the strain.

The KEGG database annotations are summarized in Figure 2C. A total of 3,168 genes were annotated, accounting for 65.18% of the genome. The number of genes involved in metabolism was the largest, with a total of 2,487, accounting for 78.5% of the total number of genes annotated in the KEGG database. A total of 342 genes were involved in carbohydrate metabolism, and glycan biosynthesis and metabolism, which may be associated with the ability of the strain to efficiently degrade macromolecular polysaccharides such as starch; this would also be consistent with the high amylase activity measured among the physiological and biochemical indicators. Many genes were involved in the metabolism of xenobiotics, such as terpenoid metabolism, polyketide metabolism, and biodegradation of xenobiotics. These metabolic pathways suggest that *P. fragi* D12, similar to other *Pseudomonas* strains, has great potential for environmental remediation.

The GO functional annotation of the *P. fragi* D12 genome is summarized in Figure 2D. A total of 3,207 genes were annotated, accounting for 65.98% of the total number of genes. The GO terms were classified into the categories Molecular Function, Biological Process, and Cellular Component. Among these categories, binding and catalytic activities, cellular processes, metabolic processes, and cellular anatomical entities (flagellum and pilin) were the most highly enriched components, which may be associated with the tolerance and cold-adaptive characteristics of *P. fragi* D12. These pathways would be important for *P. fragi* D12 to maintain a high level of metabolic capacity and enzyme production capacity. In addition, 233 annotated genes were involved in stress responses, which can also be guaranteed to survive in the face of extreme environments. Annotation of the *P. fragi* D12 strain genome using the COG database (Figure 2E) revealed that 3,858 sequences coding for amino acids of proteins had known biological functions, accounting for 79.38% of the total number of genes. Among the genes with known functions, metabolism genes accounted for the highest proportion of which 525 genes were associated with amino acid transport and metabolism. Metabolism-related genes were abundant, which may be an important reason why *P. fragi* D12 can maintain activity in extreme environments.

### 3.3. Comparative genome analysis and cold-adaptive gene mining

Through the analysis of comparative genetics, the commonality and characteristics of genes in the evolution process of strains from different sources can be clarified. For example, Kjærbølling analyzed the comparative genomes of 23 species of *Aspergillus flavus*, analyzed the evolutionary relationship between them, and screened the gene clusters of extracellular enzymes and secondary metabolites shared between them (Kjærbølling et al., 2020). Kubicek et al. (2019) selected the 12 most common *Trichoderma* species, identified and re-annotated the core genome of *Trichoderma* through comparative genomics analysis, and found that 80% of the genes also exist in other fungi. Morin et al. (2019) highlights the genetic specificity of *Glomeromycotina* by comparative genomics. Almeida et al. (2023) compared and analyzed the genomes of 4 strains of *Clostridium perfringens*, predicted potential protein target sites that can be used to construct vaccines and carried out molecular docking on the identified target sites, which is helpful for the development of new vaccines and drugs. In this experiment, in order to explore the special cold adaptation mechanism evolved by *P. fragi* D12 under long-term low-temperature stress, we conducted a comparative genetic study on five strains of *P. fragi* (A13BB, DBC, NL20W, NMC25, and NRRLB727) (Singha et al., 2017; Awolope et al., 2021; Wu J. et al., 2022) with different origins but the closest homology. The results of collinearity prove that *P. fragi* D12 has the highest similarity with *P. fragi* NMC, which also originated from low temperature, and it is speculated that it may be derived from the same ancestor. The Venn diagram of the pan-gene data set between each strain revealed that the number of unique clusters of *P. fragi* D12 was as many as 896. This indicated that *P. fragi* D12 was a newly screened *P. fragi* and may possess a unique cold-adaptation mechanism that has evolved under long-term cold-adaptive stress. The D12 strain had 505 unique genes of which metabolism-related genes (314) accounted for the largest proportion; of these genes, 23 were involved in lipid transport and metabolism, and 107 were associated with replication, transcription, and translation. Among the 112 genes involved in the cellular process, 9, 21, 29, and 19 genes were associated with extracellular structure, envelope formation, signal transduction, and defense mechanism, respectively; in addition, 21 genes had unknown functions.

As mentioned in the introduction, in order to overcome the cold living environment, low-temperature microorganisms have evolved a series of highly complex cooperative adaptation mechanisms at the cellular level, including cell membranes, biofilms, cold shock proteins, low-temperature enzymes, cryoprotectants, molecular partner and other metabolic capacities (Collins and Margesin, 2019; Pathania et al., 2022). For example, fatty acid synthase, fatty acid desaturase, branched-chain fatty acid enzyme (KAS-II/III), and fatty acid cis/trans isomerase, etc., play a role in maintaining membrane fluidity at low temperatures (Furuya et al., 2014; Heredia and Lucchesi, 2019; Yang et al., 2020); Compatible solutes, exopolysaccharides, and antifreeze proteins provide energy and antifreeze protection for low-temperature bacteria (Yang et al., 2003; Dolev et al., 2016; Fonseca et al., 2016); Cold shock proteins, RNA helicases, and molecular chaperones regulate the transcription and translation of microorganisms at low temperature (Lim et al., 2000; Budkina et al., 2020); In addition, some antioxidant enzymes (peroxide enzyme and superoxide dismutase) prevents peroxidation in microorganisms. On this basis, we screened and summarized related genes from the annotations of the whole genome of *P. fragi* D12, and initially screened 124 possible potential cold adaptation genes (Supplementary Table S1). In the genome-wide annotation of *P. fragi* D12, 46 genes associated with cell membrane fluidity were screened, including four genes involved in the unsaturated fatty acid synthesis pathway and 42 genes in the fatty acid degradation pathway. The modes of fatty acid degradation include β-oxidation, α-oxidation, ω-oxidation, and unsaturated fatty acid oxidation. These metabolic methods can increase the content of unsaturated fatty acids and reduce the average length of fatty acid chains, thereby changing the composition of lipids in cell membranes and improving the fluidity of cell membranes at low temperatures. Seven cold-shock protein genes, five RNA helicases, and ten DNA helicases were annotated; these genes assist the entangled RNA to unwind at low temperatures, thereby restoring normal transcription and translation. Twenty-six genes associated with extracellular polymer synthesis were annotated, including two genes involved in polysaccharide synthesis, eight genes associated with pilin synthesis, and sixteen genes associated with flagellin. These genes may increase the motility ability of *P. fragi* D12 and provide a stable extracellular environment at low temperatures. Twenty-five genes associated with compatible solutes were annotated in the *P. fragi* D12 genome. One gene controls several related compatible solutes, indicating that these compatible solutes are regulated simultaneously. The production of active and compatible solutes can lower the freezing point of the intracellular environment and provide a stable internal environment. Five genes associated with the ROS balance were annotated, comprising three catalases and two superoxide dismutases, which would maintain the ROS balance at low temperatures.

Nineteen unique cold-adaptive genes were screened from the *P. fragi* D12 genome (Table 3). These include genes involved in fatty acid metabolism, synthesis of extracellular polymers, and compatible solutes. It was observed that the *P. fragi* D12 genome contained a greater number of motility elements. It is speculated that, at low temperature, the relatively more abundant motility elements may contribute to the adaptability of *P. fragi* D12. The higher number of genes associated with extracellular polymers and compatible solutes provides a more stable internal and external environment for strains at low temperatures. In addition, *P. fragi* D12 contained a greater number of genes associated with the utilization of carbon and nitrogen sources, and genes associated with metabolism and lipid synthesis and transportation, which presumably enhanced the stress resistance and cold tolerance of *P. fragi* D12.

**Table 3:** Cold-adaptive genes unique to *Pseudomonas fragi* D12.

Gene id	Gene size (bp)	Gene name	Gene define
Fatty acid degradation			
D12GL001416	1,155	adh	alcohol dehydrogenase
D12GL001417	1,155	adh	alcohol dehydrogenase
D12GL001757	1,002	adh	alcohol dehydrogenase
D12GL001954	927	adh	alcohol dehydrogenase
D12GL002230	690	acd	acyl-CoA dehydrogenase
D12GL002643	1761	acd	acyl-CoA dehydrogenase
D12GL001267	819	fabG	3-oxoacyl-[acyl-carrier protein] reductase
D12GL001758	741	fabG	3-oxoacyl-[acyl-carrier protein] reductase
D12GL002446	759	fabG	3-oxoacyl-[acyl-carrier protein] reductase
D12GL002644	1,206	atoB	acetyl-CoA C-acetyltransferase
D12GL001767	843	kduD	2-dehydro-3-deoxy-D-gluconate 5-dehydrogenase
Extracellular polymer			
D12GL002916	1,038	exoZ	exopolysaccharide production protein ExoZ
D12GL000302	999	pilT	twitching motility protein PilT
D12GL002241	528	fimA	major type 1 subunit fimbrin (pilin)
D12GL003152	549	fimA	major type 1 subunit fimbrin (pilin)
D12GL004045	528	fimA	major type 1 subunit fimbrin (pilin)
Compatible solutes			
D12GL002458	1,173	thuE	trehalose/maltose transport system substrate-binding protein
D12GL002459	858	thuF/sugA	trehalose/maltose transport system permease protein
D12GL002460	843	thuG/sugB	trehalose/maltose transport system permease protein

### 3.4. Transcriptome analysis

**Figure 3:**
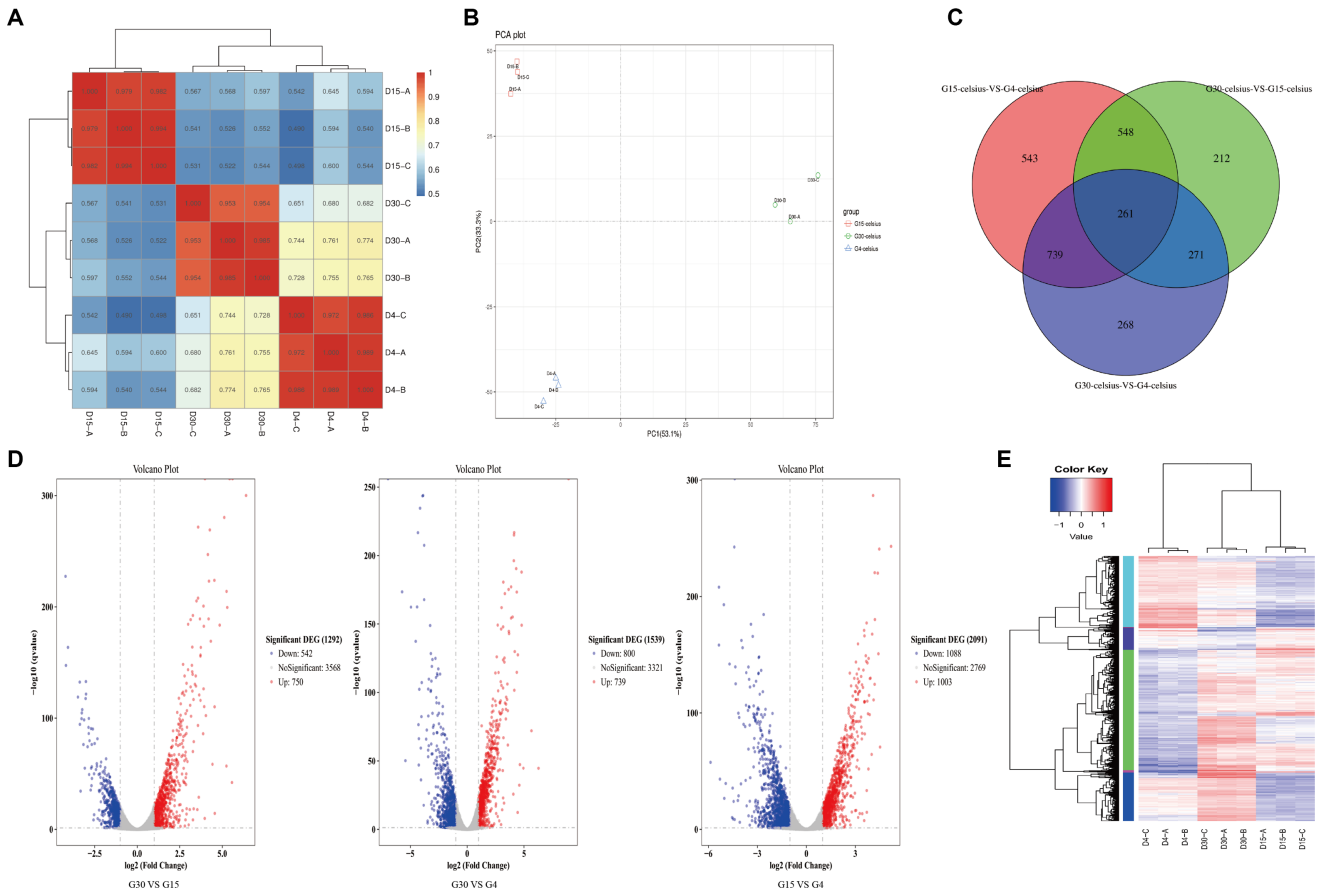
Transcriptomic information for *Pseudomonas fragi* D12. **(A)** RNA-seq correlation check heatmap; **(B)** principal component analysis scatterplot of the first and second axes; **(C)** venn diagram; **(D)** volcano plot of differentially expressed genes (DEGs); **(E)** cluster analysis dendrogram and heatmap for DEGs.

Preliminary quality analysis was performed on the raw data from transcriptome sequencing, and the quality scores were evaluated before preprocessing. The low-quality data were filtered to remove contamination and adapter sequences. The quality analysis after filtering proved that the samples were properly processed and of high quality. Alignment of the filtered sequences with the reference genome showed that most genes were detected in the reference genome. The RNA-seq correlation check heatmap (Figure 3A) and principal component analysis (Figure 3B) showed that replicate samples were similar between the same treatment groups, and samples from different treatment groups were distinctly separated, supporting the reliability of the transcriptome data. The detection results were screened according to the standard of difference significance (differential gene expression changed more than two times and *q*-value ≤0.05), and the up-regulation and down-regulation of significantly gene differential expression were counted. The results are shown in Figure 3D. A volcano plot of the DEGs revealed distinct differences between the different treatment groups. Compared with G30, G15 comprised 750 up-regulated genes and 542 down-regulated genes; compared with G4, G30 comprised 739 up-regulated genes and 800 down-regulated genes; and compared with G15, G4 comprised 1,003 up-regulated genes and 1,088 down-regulated genes.

**Figure 4:**
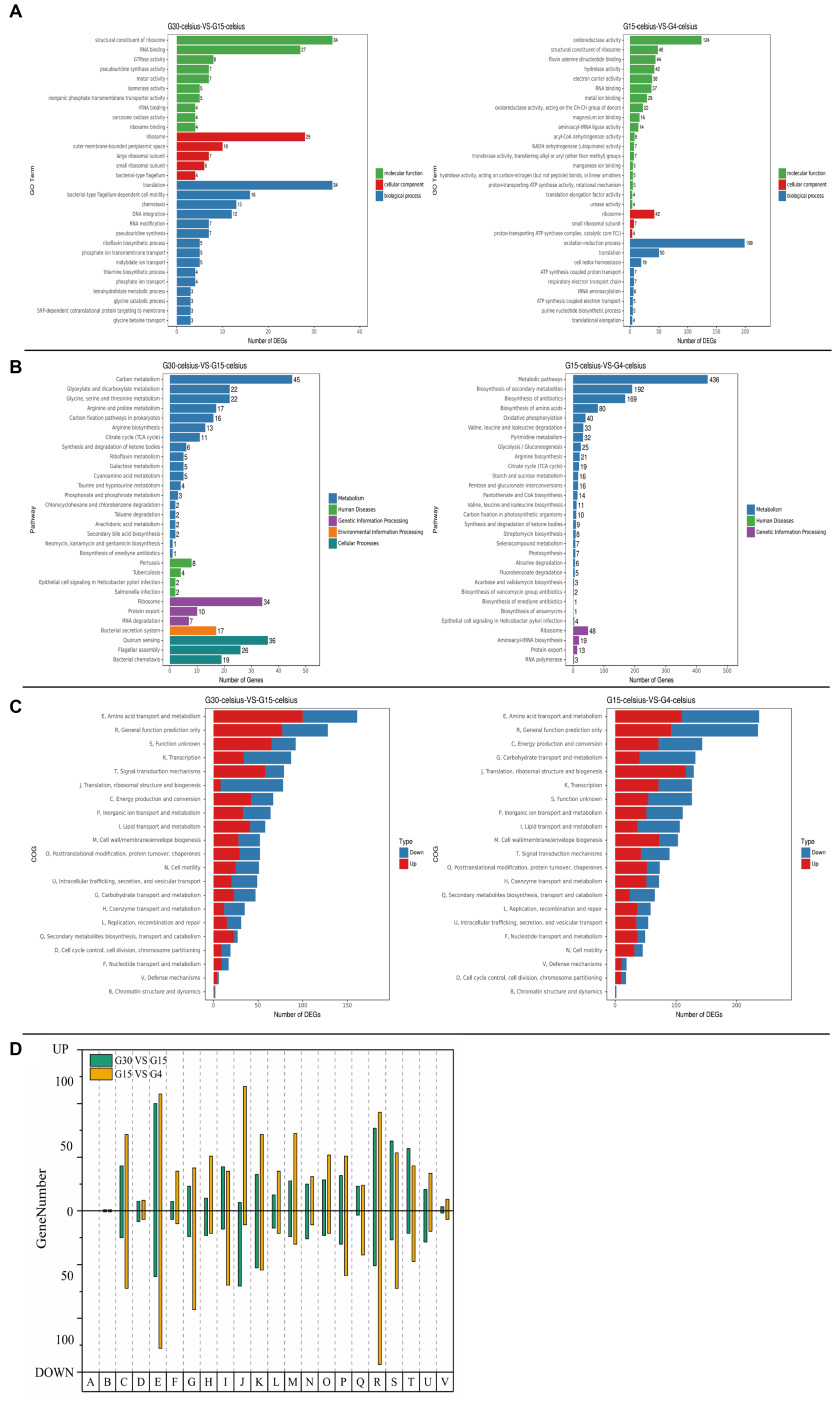
Transcriptome annotation of different comparison groups for *Pseudomonas fragi* D12. The groups were cultured at 30°C (G30), 15°C (G15), or 4°C (G4) for 2 h. Significantly differentially expressed genes (DEGs) were determined for the comparisons G30 vs. G15 and G15 vs. G4. **(A)** GO terms enriched with significantly DEGs; **(B)** KEGG pathways enriched with significantly DEGs; **(C)** COG annotation of significantly DEGs; **(D)** statistics for up-regulated and down-regulated DEGs.

A Venn diagram of the DEGs visualized the number of unique DEGs and the number of common DEGS between samples (Figure 3C). Cluster analysis of the DEGs (Figure 3E) revealed that, contrary to expectation, the same genes showed different expression patterns at 15°C and 4°C, rather than continuously high or low expression levels. This result indicated that *P. fragi* D12 exhibited different adaptation mechanisms under different degrees of temperature change. For further analysis, we temporarily discarded the G30 vs. G4 comparison group. Thus, we focused on two treatment groups, the temperature decrease from 30°C to 15°C (G30 vs. G15) and that from 15°C to 4°C (G15 vs. G4). The GO annotations of the DEGs were classified and counted. The 30 GO terms with the most significant enrichment were selected and displayed in Figure 4A, which intuitively showed the distribution of GO function terms among the DEGs. In the G30 vs. G15 comparison group, ribosome and its structural composition, RNA binding and translation-related genes accounted for the largest proportion. In the G15 vs. G4 comparison group, genes associated with redox activity accounted for the largest proportion. The most important biochemical metabolic pathways and signal transduction pathways involving the DEGs were determined from the KEGG pathway annotations (Figure 4B). For genes with significant differences in expression, the pathway annotation information was calculated. Genes associated with carbon metabolism, ribosomes, quorum sensing, and flagellar movement accounted for the majority in the G30 vs. G15 comparison group. In the G15 vs. G4 comparison group, genes associated with metabolic pathways, secondary metabolite synthesis, and ribosomes accounted for a higher proportion. To determine the specific up-regulation and down-regulation of significant DEGS in the different comparison groups, we conducted a more in-depth mining and analysis.

### 3.5. Analysis of the cold-adaptation mechanism of *Pseudomonas fragi* D12

The expression levels of cold-adaptation genes at different temperatures were further analyzed using the transcriptome data and genes with significant differences in expression were screened (Supplementary Table S2). When the temperature decreased from 30°C to 15°C (moderately low temperature), the up-regulated genes were associated with fatty acid degradation, polysaccharides, pilin, compatible solutes, and catalase. Under this temperature change, *P. fragi* D12 may maintain the fluidity of the cell membranes by shortening the average chain length of fatty acids. By increasing the expression level of the pilus protein, improving the adhesion ability, and increasing the content of extracellular polymers, the stability of the extracellular environment would be maintained. In contrast, increase in the expression of compatible solutes and catalase would maintain the stability of the intracellular environment. With further decline in the temperature from 15°C to 4°C (low temperature), most genes associated with fatty acid degradation were down-regulated, whereas the crucial genes involved in unsaturated fatty acid synthesis were up-regulated. It is speculated that *P. fragi* D12 maintains the fluidity of the cell membrane by increasing the content of unsaturated fatty acids under this temperature change. In addition to pilin synthesis, most genes associated with flagellin synthesis were up-regulated. Furthermore, the majority of genes associated with cold shock proteins, helicases, and transcription molecules were up-regulated.

**Figure 5:**
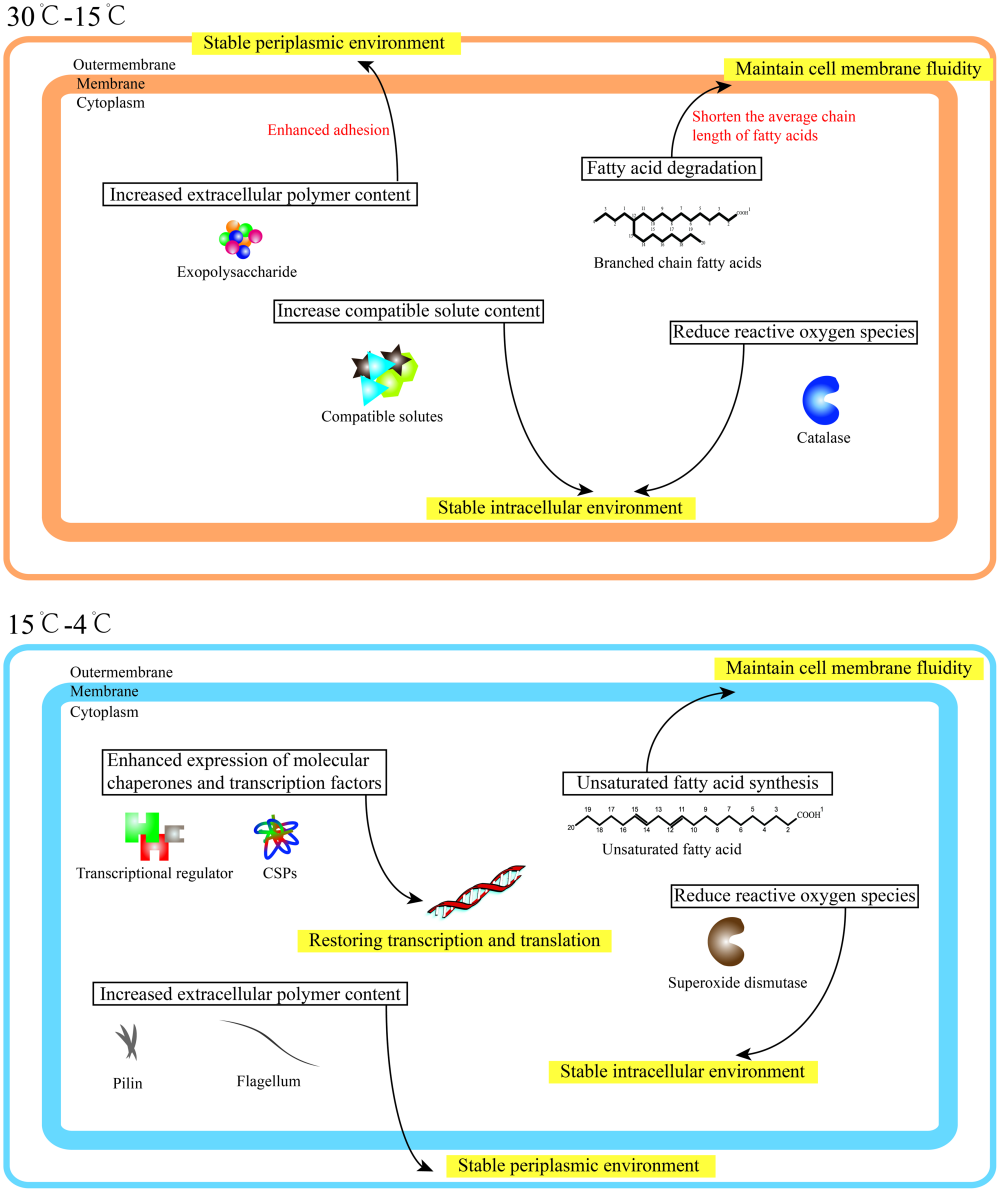
Cold-adaptation mechanism of *Pseudomonas fragi* D12.

In conclusion, when the ambient temperature decreases from 30°C to 15°C, *P. fragi* D12 maintained the stability of the internal and external environment of cells, and, at the same time, enhanced lipid metabolism and transport, and the ability to transport intracellular material, thereby improving energy metabolism at a low temperature and maintaining bacterial growth and reproduction. When the ambient temperature declined further to 4°C, *P. fragi* D12 strongly increased the expression levels of ribosomes, molecular chaperones, transcription factors, and other related genes (Figures 4C,D). We speculate that, at this time, the mRNA of bacteria folds at a low temperature, and thus normal transcription and translation cannot proceed smoothly (Kelley et al., 1997). The bacteria must increase the transcription level of the mentioned genes and restore normal metabolism. Of course, it is also necessary to maintain the stability of the internal and external environment of the cell. Some researchers have previously found changes in some genes at different degrees of low temperatures. For example, Firdaus-Raih et al. (2018) analyzed nine antifreeze proteins (AFPs) and found that nine different AFPs were able to survive under different extreme cold conditions and exposure times. Expression to ensure that cells can always be protected against freezing in the fluctuating low-temperature range, not all antifreeze proteins are highly expressed as the temperature decreases. In addition, the psychrophilic yeast Glaciozyma antarctica PI12 grown at 12°C was subjected to cold shock at 0°C and − 12°C respectively, and it was found that differentially expressed genes were different under different cold degrees. For example, three transcription factors were up-regulated at −12°C to promote transcription, there is no need for these three genes to be up-regulated at 0°C (Wong et al., 2019). But no previous researchers have come up with an overall theoretical account of this. It is easy to understand that, just as we do not need to wear down jackets in early autumn, bacteria have different adaptation mechanisms to different degrees of low temperature. Here we propose, it is that bacteria can adapt to moderate changes in ambient temperature as long as they increase their metabolism and maintain the fluidity of the cell membrane to ensure normal exchange of substances. Under more extreme temperature changes, the genetic material of the bacteria will change, which requires adoption of more complex mechanisms to increase the expression of genes such as molecular chaperones and transcription factors, and restore the normal transcription and translation of genetic material (Figure 5). In this manner, normal growth and reproduction can resume.

It is worth mentioning that, among the unique genes differentially expressed in *P. fragi* D12, three genes were significantly up-regulated at both 15°C and 4°C: D12GL002241 (type 1 fimbria component protein), D12GL002240 (P pilus assembly protein, chaperone PapD), and D12GL002239 (Outer membrane usher protein FimD/PapC). Among them, D12GL002241 has been discovered in the previous excavation of unique cold-adaptive genes, and D12GL002240 and D12GL002239 may be further discovered here as molecular chaperones of pilin synthesis. These genes are all associated with pilin synthesis. We speculate that, under long-term cold-adaptive stress, *P. fragi* D12 may enhance bacterial adhesion by increasing the protein content of the pilus, thereby increasing the production of extracellular polymers and formation of the outer cell membrane. These changes will enable maintenance of the stability of the extracellular environment, reduction in the freezing point of the surrounding environment, and resistance to changes in temperature.

## 4. Conclusion

A comparative genomics analysis was conducted and 19 unique cold-adaptive genes were screened in the genome of *P. fragi* D12. Three of the genes encoding pilus proteins were significantly up-regulated at low temperatures, which is speculated to be an evolutionary adaptation unique to *P. fragi* D12. *Pseudomonas fragi* D12 showed different responses to different degrees of temperature reduction. However, maintaining the stability of the internal and external environment, and the fluidity of cell membranes at low temperatures consistently played a crucial role in energy metabolism and material exchange. Under a more extreme low temperature, the cells enhanced the expression of molecular chaperones and transcription factors to restore the normal transcription and translation of genetic material, thereby maintaining its growth and reproduction. The results further improved and supplemented the cold-adaptation mechanism of microorganisms, while providing a new direction for studying the cold-adaptation mechanism of microorganisms. At present, more research on pilin is related to the pathogenicity of microorganisms. In future research, we plan to associate pilin with the cold-adaptation mechanism based on their key role in biofilms.

## Data availability statement

The datasets presented in this study can be found in online repositories. The names of the repository/repositories and accession number(s) can be found at: https://www.ncbi.nlm.nih.gov/genbank/, CP104861-CP104862.

## Author contributions

CB: formal analysis and writing. JM, NZ, and YZ: validation. YL and JS: resource. GC: supervision. SZ and HC: investigation and review. All authors contributed to the article and approved the submitted version.

## Funding

This work was supported by the Chinese Academy of Sciences Strategic Pilot Science and Technology Project (XDA28020400), the Natural Science Foundation of Science and Technology Department of Jilin Province (grant number 20220101334JC), the Analysis of Microbial Diversity of Low Temperature Straw Degradation and Production and Demonstration of Stalk Decomposing Agent project from the Jilin Provincial Science and Technology Depatmet (20200103155SF), and the Key Projects of the Jilin Province Science and Technology Development Plan (grant number 20210203117SF).

## Conflict of interest

The authors declare that the research was conducted in the absence of any commercial or financial relationships that could be construed as a potential conflict of interest.

## Publisher’s note

All claims expressed in this article are solely those of the authors and do not necessarily represent those of their affiliated organizations, or those of the publisher, the editors and the reviewers. Any product that may be evaluated in this article, or claim that may be made by its manufacturer, is not guaranteed or endorsed by the publisher.

## Footnotes

^1^https://github.com/PacificBiosciences/pbdagcon

^2^http://sourceforge.net/projects/wgs-assembler/files/wgs-assembler/wgs-8.3/

^3^https://www.broadinstitute.org/gatk/

^4^http://www.cbcb.umd.edu/software/glimmer/

^5^http://www.cbs.dtu.dk/services/RNAmmer/

^6^http://gtrnadb.ucsc.edu/

^7^http://rfam.sanger.ac.uk/

^8^http://tandem.bu.edu/trf/trf.html

^9^http://www5.esu.edu/cpsc/bioinfo/software/GIST/

^10^http://phast.wishartlab.com/

^11^https://crisprcas.i2bc.paris-saclay.fr/CrisprCasFinder/

^12^http://mummer.sourceforge.net/

^13^http://weizhongli-lab.org/cd-hit/

^14^http://treesoft.svn.sourceforge.net/viewvc/treesoft/trunk/treebest/
