# Supplementary material for: Mining of key genes for cold adaptation from Pseudomonas fragi D12 and analysis of its cold-adaptation mechanism
Source: Front Microbiol. 2023 Jul 6;14:1215837. doi: 10.3389/fmicb.2023.1215837 (PMC10358777; doi:10.3389/fmicb.2023.1215837)
Supplement: Supplementary file 1 [file Data_Sheet_1.docx]

**Supplemental Tables**

Supplemental Table 1 Cold-adaptive genes of *P.fragi* D12

| Gene  id | Gene  size(bp) | Gene  name | Gene  define |
| --- | --- | --- | --- |
| Cell membrane fluidity-related genes | | | |
| D12GL004696 | 1185 | SCD/desC | stearoyl-CoA desaturase (Delta-9 desaturase) |
| D12GL001455 | 606 | tesA | acyl-CoA thioesterase I |
| D12GL000654 | 870 | tesB | acyl-CoA thioesterase II |
| D12GL004731 | 402 | yciA | acyl-CoA thioesterase YciA |
| D12GL001416 | 1155 | adh | alcohol dehydrogenase |
| D12GL001417 | 1155 | adh | alcohol dehydrogenase |
| D12GL001757 | 1002 | adh | alcohol dehydrogenase |
| D12GL001954 | 927 | adh | alcohol dehydrogenase |
| D12GL002353 | 1011 | adh | alcohol dehydrogenase |
| D12GL003753 | 1113 | frmA/ADH5/adhC | S-(hydroxymethyl)glutathione alcohol dehydrogenase |
| D12GL004357 | 1491 | ALDH | aldehyde dehydrogenase |
| D12GL000417 | 1779 | ACADS/bcd | butyryl-CoA dehydrogenase |
| D12GL002784 | 1128 | ACADS/bcd | butyryl-CoA dehydrogenase |
| D12GL002795 | 1143 | ACADS/bcd | butyryl-CoA dehydrogenase |
| D12GL001864 | 1185 | ACADM/acd | acyl-CoA dehydrogenase |
| D12GL002146 | 1191 | ACADM/acd | acyl-CoA dehydrogenase |
| D12GL002147 | 1143 | ACADM/acd | acyl-CoA dehydrogenase |
| D12GL002383 | 1773 | ACADM/acd | acyl-CoA dehydrogenase |
| D12GL002643 | 1761 | ACADM/acd | acyl-CoA dehydrogenase |
| D12GL002682 | 1749 | ACADM/acd | acyl-CoA dehydrogenase |
| D12GL002741 | 1164 | ACADM/acd | acyl-CoA dehydrogenase |
| D12GL003392 | 1206 | ACADM/acd | acyl-CoA dehydrogenase |
| D12GL004713 | 1182 | GCDH/gcdH | glutaryl-CoA dehydrogenase |
| D12GL001875 | 1152 | atoB | acetyl-CoA C-acetyltransferase |
| D12GL001879 | 1221 | atoB | acetyl-CoA C-acetyltransferase |
| D12GL001901 | 1182 | atoB | acetyl-CoA C-acetyltransferase |
| D12GL002644 | 1206 | atoB | acetyl-CoA C-acetyltransferase |
| D12GL002699 | 1182 | atoB | acetyl-CoA C-acetyltransferase |
| D12GL002785 | 1194 | atoB | acetyl-CoA C-acetyltransferase |
| D12GL004255 | 1275 | atoB | acetyl-CoA C-acetyltransferase |
| D12GL001363 | 1155 | fadA/fadI | acetyl-CoA acyltransferase |
| D12GL001295 | 690 | paaF/echA | enoyl-CoA hydratase |
| D12GL001872 | 765 | paaF/echA | enoyl-CoA hydratase |
| D12GL002139 | 786 | paaF/echA | enoyl-CoA hydratase |
| D12GL002169 | 906 | paaF/echA | enoyl-CoA hydratase |
| D12GL002781 | 1110 | paaF/echA | enoyl-CoA hydratase |
| D12GL002783 | 774 | paaF/echA | enoyl-CoA hydratase |
| D12GL003356 | 813 | paaF/echA | enoyl-CoA hydratase |
| D12GL001362 | 2148 | fadB | enoyl-CoA hydratase |
| D12GL002148 | 1689 | ACSL/fadD | long-chain acyl-CoA synthetase |
| D12GL003657 | 1692 | ACSL/fadD | long-chain acyl-CoA synthetase |
| D12GL003658 | 1689 | ACSL/fadD | long-chain acyl-CoA synthetase |
| D12GL000174 | 1149 | rubB/alkT | rubredoxin---NAD^+^ reductase |
| D12GL000175 | 168 | rubB/alkT | rubredoxin---NAD^+^ reductase |
| D12GL001233 | 2448 | fadE | acyl-CoA dehydrogenase |
| D12GL003545 | 1149 | yiaY | alcohol dehydrogenase |
| Cold shock protein and helicase gene | | | |
| D12GL001010 | 210 | cspA | cold shock protein (beta-ribbon, CspA family) |
| D12GL001555 | 279 | cspA | cold shock protein (beta-ribbon, CspA family) |
| D12GL002335 | 438 | cspA | cold shock protein (beta-ribbon, CspA family) |
| D12GL003025 | 213 | cspA | cold shock protein (beta-ribbon, CspA family) |
| D12GL003520 | 603 | cspA | cold shock protein (beta-ribbon, CspA family) |
| D12GL003701 | 213 | cspA | cold shock protein (beta-ribbon, CspA family) |
| D12GL003792 | 186 | cspA | cold shock protein (beta-ribbon, CspA family) |
| D12GL000323 | 1854 | rhlE | ATP-dependent RNA helicase RhlE |
| D12GL000650 | 1338 | rhlE | ATP-dependent RNA helicase RhlE |
| D12GL000355 | 1386 | dbpA | ATP-independent RNA helicase DbpA |
| D12GL001330 | 1674 | deaD | ATP-dependent RNA helicase DeaD |
| D12GL003294 | 1347 | srmB | ATP-dependent RNA helicase SrmB |
| D12GL003890 | 1497 | rhlB | ATP-dependent RNA helicase RhlB |
| D12GL000130 | 1977 | rep | ATP-dependent DNA helicase Rep |
| D12GL000170 | 2118 | recG | ATP-dependent DNA helicase RecG |
| D12GL001024 | 2145 | dinG | ATP-dependent DNA helicase DinG |
| D12GL003279 | 2130 | recQ | ATP-dependent DNA helicase RecQ |
| D12GL004537 | 1971 | recQ | ATP-dependent DNA helicase RecQ |
| D12GL000629 | 2520 | hrpB | ATP-dependent helicase HrpB |
| D12GL001045 | 2847 | hepA | ATP-dependent helicase HepA |
| D12GL003309 | 3933 | hrpA | ATP-dependent helicase HrpA |
| D12GL003004 | 2697 |  | putative DNA primase/helicase |
| D12GL004365 | 1401 | dnaB | replicative DNA helicase |
| D12GL004754 | 2184 | uvrD/pcrA | DNA helicase II |
| D12GL003512 | 1059 | ruvB | holliday junction DNA helicase RuvB |
| D12GL003513 | 609 | ruvA | holliday junction DNA helicase RuvA |
| Extracellular polymer synthesis related genes | | | |
| D12GL002916 | 1038 | exoZ | exopolysaccharide production protein ExoZ |
| D12GL001641 | 1923 | epsC | polysaccharide biosynthesis protein EpsC |
| D12GL000314 | 540 | pilI | twitching motility protein PilI |
| D12GL000315 | 2058 | pilJ | twitching motility protein PilJ |
| D12GL000316 | 5862 | pilL | type IV pili sensor histidine kinase and response regulator |
| D12GL000317 | 453 | chpC | chemosensory pili system protein ChpC |
| D12GL002241 | 528 | fimA | major type 1 subunit fimbrin (pilin) |
| D12GL002659 | 402 | fimA | major type 1 subunit fimbrin (pilin) |
| D12GL003152 | 549 | fimA | major type 1 subunit fimbrin (pilin) |
| D12GL004045 | 528 | fimA | major type 1 subunit fimbrin (pilin) |
| D12GL003242 | 1728 | fliC | flagellin |
| D12GL003567 | 321 | flgM | negative regulator of flagellin synthesis FlgM |
| D12GL003244 | 1581 | flgL | flagellar hook-associated protein 3 FlgL |
| D12GL003245 | 2025 | flgK | flagellar hook-associated protein 1 FlgK |
| D12GL003246 | 1236 | flgJ | flagellar protein FlgJ |
| D12GL003247 | 1125 | flgI | flagellar P-ring protein precursor FlgI |
| D12GL003248 | 696 | flgH | flagellar L-ring protein precursor FlgH |
| D12GL003249 | 786 | flgG | flagellar basal-body rod protein FlgG |
| D12GL003250 | 741 | flgF | flagellar basal-body rod protein FlgF |
| D12GL003560 | 1320 | flgE | flagellar hook protein FlgE |
| D12GL003561 | 726 | flgD | flagellar basal-body rod modification protein FlgD |
| D12GL003562 | 429 | flgC | flagellar basal-body rod protein FlgC |
| D12GL003563 | 408 | flgB | flagellar basal-body rod protein FlgB |
| D12GL003566 | 621 | flgA | flagella basal body P-ring formation protein FlgA |
| D12GL003567 | 321 | flgM | negative regulator of flagellin synthesis FlgM |
| D12GL003568 | 468 | flgN | flagella synthesis protein FlgN |
| Compatible solute-related genes | | | |
| D12GL000027 | 1959 | betT/betS | choline/glycine/proline betaine transport protein |
| D12GL000364 | 1995 | betT/betS | choline/glycine/proline betaine transport protein |
| D12GL000365 | 1179 | proV | glycine betaine/proline transport system ATP-binding protein |
| D12GL001227 | 831 | proV | glycine betaine/proline transport system ATP-binding protein |
| D12GL000366 | 846 | proW | glycine betaine/proline transport system permease protein |
| D12GL001226 | 852 | proW | glycine betaine/proline transport system permease protein |
| D12GL000367 | 1050 | proX | glycine betaine/proline transport system substrate-binding protein |
| D12GL001225 | 1023 | proX | glycine betaine/proline transport system substrate-binding protein |
| D12GL001503 | 819 | proX | glycine betaine/proline transport system substrate-binding protein |
| D12GL000381 | 1293 | gbcA | glycine betaine catabolism A |
| D12GL000382 | 1113 | gbcB | glycine betaine catabolism B |
| D12GL001703 | 1410 | cycA | D-serine/D-alanine/glycine transporter |
| D12GL002454 | 1251 | cycA | D-serine/D-alanine/glycine transporter |
| D12GL004739 | 1422 | TC/AGCS | alanine or glycine:cation symporter, AGCS family |
| D12GL000009 | 1506 | proP | proline/betaine transporter |
| D12GL003067 | 1338 | proP | MFS transporter, MHS family, proline/betaine transporter |
| D12GL004730 | 1290 | proP | proline/betaine transporter |
| D12GL000369 | 1104 | gbdR | glycine betaine-responsive activator |
| D12GL002705 | 1026 | gbdR | glycine betaine-responsive activator |
| D12GL002458 | 1173 | thuE | trehalose/maltose transport system substrate-binding protein |
| D12GL002459 | 858 | thuF/sugA | trehalose/maltose transport system permease protein |
| D12GL002460 | 843 | thuG/sugB | trehalose/maltose transport system permease protein |
| D12GL002272 | 834 | smoG/mtlG | sorbitol/mannitol transport system permease protein |
| D12GL002273 | 927 | smoF/mtlF | sorbitol/mannitol transport system permease protein |
| D12GL002274 | 1311 | smoE/mtlE | sorbitol/mannitol transport system substrate-binding protein |
| Reactive oxygen balance related genes | | | |
| D12GL000526 | 1449 | KatE/CAT/catB/srpA | catalase |
| D12GL000685 | 1542 | KatE/CAT/catB/srpA | catalase |
| D12GL002048 | 1101 | KatE/CAT/catB/srpA | catalase |
| D12GL001111 | 597 | SOD2 | superoxide dismutase |
| D12GL003989 | 657 | SOD2 | superoxide dismutase |

Supplemental Table 2 Analysis of differential expression of cold-adaptive genes in different comparison groups

| Gene id | log2FoldChange | Regulation | Gene difine |
| --- | --- | --- | --- |
| G30 VS G15 | | | |
| Cell membrane fluidity | | | |
| D12GL004696 | -1.010349443 | Down | stearoyl-CoA desaturase (Delta-9 desaturase) |
| D12GL001954 | 1.112016619 | Ups | alcohol dehydrogenase |
| D12GL002353 | 3.717928028 | Ups | alcohol dehydrogenase |
| D12GL002784 | 1.555415084 | Ups | butyryl-CoA dehydrogenase |
| D12GL001864 | 1.255806081 | Ups | acyl-CoA dehydrogenase |
| D12GL004713 | 1.502051174 | Ups | glutaryl-CoA dehydrogenase |
| D12GL002699 | 1.470358481 | Ups | acetyl-CoA C-acetyltransferase |
| D12GL002785 | 2.055709693 | Ups | acetyl-CoA C-acetyltransferase |
| D12GL004255 | 1.687298114 | Ups | acetyl-CoA C-acetyltransferase |
| D12GL002781 | -1.172967977 | Down | enoyl-CoA hydratase |
| D12GL002783 | 2.806627813 | Ups | enoyl-CoA hydratase |
| D12GL003658 | 2.282362249 | Ups | long-chain acyl-CoA synthetase |
| D12GL001233 | 1.7198602 | Ups | acyl-CoA dehydrogenase |
| Cold shock proteins and helicases | | | |
| D12GL001555 | 1.55987644 | Ups | cold shock protein (beta-ribbon, CspA family) |
| D12GL003520 | -1.162009382 | Down | cold shock protein (beta-ribbon, CspA family) |
| D12GL003701 | -2.165831381 | Down | cold shock protein (beta-ribbon, CspA family) |
| D12GL003792 | 1.347594015 | Ups | cold shock protein (beta-ribbon, CspA family) |
| D12GL001045 | -1.213711876 | Down | ATP-dependent helicase HepA |
| D12GL004365 | -1.158786613 | Down | replicative DNA helicase |
| D12GL004754 | -1.106726107 | Down | DNA helicase II / ATP-dependent DNA helicase PcrA |
| Extracellular polymers | | | |
| D12GL002916 | 1.87949292 | Ups | exopolysaccharide production protein ExoZ |
| D12GL002241 | 4.541156802 | Ups | major type 1 subunit fimbrin (pilin) |
| D12GL003152 | -1.640512872 | Down | major type 1 subunit fimbrin (pilin) |
| D12GL003242 | 3.739442486 | Ups | flagellin |
| D12GL003246 | -2.265092936 | Down | flagellar protein FlgJ |
| D12GL003247 | -3.257597829 | Down | flagellar P-ring protein precursor FlgI |
| D12GL003248 | -2.863833239 | Down | flagellar L-ring protein precursor FlgH |
| D12GL003249 | -2.803221935 | Down | flagellar basal-body rod protein FlgG |
| D12GL003250 | -4.069055638 | Down | flagellar basal-body rod protein FlgF |
| D12GL003560 | -1.557407155 | Down | flagellar hook protein FlgE |
| D12GL003561 | -2.536068008 | Down | flagellar basal-body rod modification protein |
| D12GL003562 | -2.732567349 | Down | flagellar basal-body rod protein FlgC |
| D12GL003563 | -3.161768816 | Down | flagellar basal-body rod protein FlgB |
| D12GL003566 | -3.123990361 | Down | flagella basal body P-ring formation protein |
| Compatible solutes | | | |
| D12GL000365 | 1.29254807 | Ups | glycine betaine/proline transport system ATP-binding protein |
| D12GL001227 | 1.507909185 | Ups | glycine betaine/proline transport system ATP-binding protein |
| D12GL000366 | 1.524009651 | Ups | glycine betaine/proline transport system permease protein |
| D12GL000367 | 1.798281621 | Ups | glycine betaine/proline transport system substrate-binding protein |
| D12GL001503 | 1.127660045 | Ups | glycine betaine/proline transport system substrate-binding protein |
| D12GL000381 | 1.153892098 | Ups | glycine betaine catabolism A |
| D12GL000382 | 1.159252119 | Ups | glycine betaine catabolism B |
| D12GL001703 | 1.443867464 | Ups | D-serine/D-alanine/glycine transporter |
| D12GL004739 | 1.321851778 | Ups | alanine or glycine:cation symportery |
| D12GL000009 | 1.95753217 | Ups | proline/betaine transporter |
| D12GL004730 | -1.15058983 | Down | proline/betaine transporter |
| D12GL000369 | 1.52132737 | Ups | AraC family transcriptional regulator, glycine betaine-responsive activator |
| D12GL002273 | -1.092800416 | Down | sorbitol/mannitol transport system permease protein |
| Reactive oxygen | | | |
| D12GL000526 | 3.573043821 | Ups | catalase |
| G15 VS G4 |  |  |  |
| Cell membrane fluidity | | | |
| D12GL004696 | 1.1169073 | Ups | stearoyl-CoA desaturase (Delta-9 desaturase) |
| D12GL001455 | -1.598426731 | Down | acyl-CoA thioesterase I |
| D12GL001416 | -1.36157973 | Down | alcohol dehydrogenase |
| D12GL001417 | -1.834421293 | Down | alcohol dehydrogenase |
| D12GL001757 | -1.230335613 | Down | alcohol dehydrogenase |
| D12GL001954 | -2.309690236 | Down | alcohol dehydrogenase |
| D12GL002353 | -2.00576835 | Down | alcohol dehydrogenase |
| D12GL002784 | -2.581291183 | Down | butyryl-CoA dehydrogenase |
| D12GL002795 | -2.290047505 | Down | butyryl-CoA dehydrogenase |
| D12GL001864 | -2.422542175 | Down | acyl-CoA dehydrogenase |
| D12GL002643 | -1.64943081 | Down | acyl-CoA dehydrogenase |
| D12GL002682 | -2.240625429 | Down | acyl-CoA dehydrogenase |
| D12GL002741 | -1.501596656 | Down | acyl-CoA dehydrogenase |
| D12GL004713 | -1.370333191 | Down | glutaryl-CoA dehydrogenase |
| D12GL001879 | -1.019478781 | Down | acetyl-CoA C-acetyltransferase |
| D12GL002644 | -1.566138873 | Down | acetyl-CoA C-acetyltransferase |
| D12GL002699 | 1.330979873 | Ups | acetyl-CoA C-acetyltransferase |
| D12GL002785 | -1.065203224 | Down | acetyl-CoA C-acetyltransferase |
| D12GL004255 | -3.679345477 | Down | acetyl-CoA C-acetyltransferase |
| D12GL001295 | -1.557122084 | Down | enoyl-CoA hydratase |
| D12GL002139 | -2.073712886 | Down | enoyl-CoA hydratase |
| D12GL002783 | -1.64921348 | Down | enoyl-CoA hydratase |
| D12GL002148 | 1.1169073 | Ups | long-chain acyl-CoA synthetase |
| D12GL003658 | -1.598426731 | Down | long-chain acyl-CoA synthetase |
| D12GL001233 | -1.36157973 | Down | acyl-CoA dehydrogenase |
| Cold shock proteins and helicases | | | |
| D12GL003025 | 1.99219775 | Ups | cold shock protein (beta-ribbon, CspA family) |
| D12GL003792 | -1.035205343 | Down | cold shock protein (beta-ribbon, CspA family) |
| D12GL000650 | 1.42149757 | Ups | ATP-dependent RNA helicase RhlE |
| D12GL000355 | 1.056692737 | Ups | ATP-independent RNA helicase DbpA |
| D12GL001330 | 1.377597275 | Ups | ATP-dependent RNA helicase DeaD |
| D12GL003294 | 1.270513618 | Ups | ATP-dependent RNA helicase SrmB |
| D12GL003890 | 1.492857071 | Ups | ATP-dependent RNA helicase RhlB |
| D12GL000170 | 1.12076292 | Ups | ATP-dependent DNA helicase RecG |
| D12GL001024 | 1.44702579 | Ups | ATP-dependent DNA helicase DinG |
| D12GL004754 | 1.427398387 | Ups | DNA helicase II / ATP-dependent DNA helicase PcrA |
| Extracellular polymers | | | |
| D12GL002916 | -1.249644458 | Down | exopolysaccharide production protein ExoZ |
| D12GL000315 | 1.078637037 | Ups | twitching motility protein PilJ |
| D12GL002241 | 4.468920905 | Ups | major type 1 subunit fimbrin (pilin) |
| D12GL002659 | -2.27755247 | Down | major type 1 subunit fimbrin (pilin) |
| D12GL003152 | 2.280662252 | Ups | major type 1 subunit fimbrin (pilin) |
| D12GL003567 | -1.812083666 | Down | negative regulator of flagellin synthesis FlgM |
| D12GL003245 | 1.215999078 | Ups | flagellar hook-associated protein 1 FlgK |
| D12GL003247 | 1.560110811 | Ups | flagellar P-ring protein precursor FlgI |
| D12GL003248 | 1.414282266 | Ups | flagellar L-ring protein precursor FlgH |
| D12GL003249 | 1.416075089 | Ups | flagellar basal-body rod protein FlgG |
| D12GL003250 | 1.614513263 | Ups | flagellar basal-body rod protein FlgF |
| D12GL003560 | 2.049172735 | Ups | flagellar hook protein FlgE |
| D12GL003561 | 2.200852299 | Ups | flagellar basal-body rod modification protein FlgD |
| D12GL003562 | 2.198837657 | Ups | flagellar basal-body rod protein FlgC |
| D12GL003563 | 2.025573965 | Ups | flagellar basal-body rod protein FlgB |
| D12GL003567 | -1.812083666 | Down | negative regulator of flagellin synthesis FlgM |
| Compatible solutes | | | |
| D12GL000364 | -1.057003587 | Down | choline/glycine/proline betaine transport protein |
| D12GL001227 | -1.607499226 | Down | glycine betaine/proline transport system ATP-binding protein |
| D12GL001226 | -1.093478217 | Down | glycine betaine/proline transport system permease protein |
| D12GL001503 | -1.936536266 | Down | glycine betaine/proline transport system substrate-binding protein |
| D12GL000382 | -1.145444524 | Down | glycine betaine catabolism B |
| D12GL002454 | -1.330983995 | Down | D-serine/D-alanine/glycine transporter |
| D12GL000009 | -3.125303283 | Down | proline/betaine transporter |
| D12GL000369 | -1.37258213 | Down | AraC family transcriptional regulator, glycine betaine-responsive activator |
| D12GL002458 | -2.202186462 | Down | trehalose/maltose transport system substrate-binding protein |
| D12GL002459 | -1.767161476 | Down | trehalose/maltose transport system permease protein |
| D12GL002272 | -1.88706234 | Down | sorbitol/mannitol transport system permease protein |
| D12GL002273 | -2.095399588 | Down | sorbitol/mannitol transport system permease protein |
| D12GL000364 | -1.057003587 | Down | choline/glycine/proline betaine transport protein |
| Reactive oxygen | | | |
| D12GL000526 | -2.76116496 | Down | catalase |
| D12GL000685 | -1.661662759 | Down | catalase |
| D12GL002048 | -1.291204843 | Down | catalase |
| D12GL001111 | 1.359062355 | Ups | superoxide dismutase |
| D12GL003989 | -1.390882714 | Down | superoxide dismutase |
